# Evaluating the implementation of the Diabetes Body Project in a clinical cohort of Australian adolescents with type 1 diabetes

**DOI:** 10.3389/fendo.2026.1958711

**Published:** 2026-09-28

**Authors:** Emma L. Fisher, Kirrilly M. Pursey, Lucy Leigh, Bruce R. King, Carmel E. Smart

**Affiliations:** 1 Paediatric Endocrinology and Diabetes, John Hunter Children’s Hospital, New Lambton Heights, NSW, Australia; 2 School of Health Sciences, College of Health Medicine and Wellbeing, The University of Newcastle, Callaghan, NSW, Australia; 3 Hunter Medical Research Institute, New Lambton Heights, NSW, Australia; 4 School of Medicine and Public Health, College of Health Medicine and Wellbeing, The University of Newcastle, Callaghan, NSW, Australia

**Keywords:** adolescents, eating disorder, pilot, prevention, type 1 diabetes

## Abstract

**Introduction:**

Adolescents with type 1 diabetes (T1D) are at increased risk of disordered eating behaviors (DEB) and eating disorders (ED) compared to their peers without T1D, contributing to increased diabetes-related complications, and elevated mortality risk. Despite this, there is a lack of targeted ED prevention interventions specifically designed for adolescents with T1D, within routine clinical care. The Diabetes Body Project (DBP) has demonstrated promising feasibility, acceptability, and reduction of DEB in young adults; however, its application in younger adolescents and real-world clinical settings has not been tested.

**Objective:**

This study aims to extend previous DBP research by evaluating its implementation within a real-world Australian clinical service. The study will assess feasibility and acceptability and explore preliminary efficacy in relation to DEB and glycaemic control in an exclusively adolescent population, including a novel parent/caregiver component.

**Methods:**

This protocol describes a pilot study that will recruit 24 female adolescents with T1D from the John Hunter Children’s Hospital (JHCH) Diabetes Service in New South Wales, Australia, along with up to 24 parents/caregivers. Adolescents will participate in a 6-week virtually delivered manualised cognitive dissonance based DBP program, delivered in a group format across four cohorts of 6 adolescents. In addition, two group sessions will be developed and delivered virtually to parents/caregivers for each cohort. Outcomes will be assessed at baseline, post-intervention and at 3 and 6 months follow up. Feasibility will be evaluated through recruitment, retention, attendance, between-session activity completion and parent/caregiver uptake. Acceptability will be assessed via participant and caregiver satisfaction surveys. Preliminary efficacy will be explored through changes in disordered eating behaviors (Diabetes Eating Problem Survey–Revised), body appreciation (BAS-2), diabetes distress (PAID-T), and glycaemic outcomes.

**Discussion:**

This study will evaluate the implementation of an adapted DBP intervention for adolescents with T1D within a real-world clinical service. By incorporating a novel parent/caregiver component and targeting the adolescent population, the study addresses key gaps in current literature. Findings may inform the design of future larger-scale trials and support the integration of ED prevention strategies into routine pediatric diabetes care.

## Introduction

Adolescents with type 1 diabetes (T1D) are at increased risk of developing disordered eating behaviors (DEB) and consequently eating disorders (ED) compared to their peers without T1D (1). This increased vulnerability may be influenced by diabetes-specific factors, including the daily demands of diabetes management, the effects of insulin on weight and hunger, and diabetes-related family conflict (2). DEB encompasses disturbed thoughts and behaviors relating to weight and shape that do not meet formal DSM-5 diagnostic ED criteria (3). Insulin omission is a specific DEB associated with T1D which can lead to life-threatening diabetic ketoacidosis (DKA) (4). Females, adolescents and those experiencing body dissatisfaction or diabetes related distress are at highest risk for insulin omission (5). Co-occurring T1D and ED is associated with increased risk of diabetes specific complications, including DKA and early-onset kidney disease, resulting in a threefold increase in premature mortality (6–8).

Globally the prevalence of DEB in this population has been reported to be between ~25-48% (9–12). Early detection and management of DEB are vital for prevention of ED, which has a peak onset in adolescents between ages 14–19 years (1, 13). Childhood and adolescent years are *recognized* as critical periods for early detection and intervention, with T1D guidelines recommending disordered eating (DE) screening from 10–12 years (14). Despite the *recognized* burden of DEB in young people with T1D, recent systematic reviews have highlighted the limited evidence available, particularly for interventions targeting younger adolescents, involving parents or caregivers, or evaluating implementation within routine clinical practice (15, 16).

Recent international studies provide some insights into potential intervention models for DEB in T1D. The Diabetes Body Project (DBP), developed in Norway, is a manualised cognitive dissonance-based ED prevention intervention that can be delivered virtually and has demonstrated good feasibility, acceptability and reductions in disordered eating symptoms among females aged 14–35 years in multicenter RCT studies (17–19). However, recruitment to DBP studies via social media has been unsuccessful in engaging adolescents (17, 18) highlighting the need to further evaluate the acceptability and preliminary efficacy of the program within this population in a clinical setting.

Parental involvement in the prevention of ED in T1D represents another important gap in the literature. Interventions involving both adolescents and parents have demonstrated greater effectiveness in reducing ED risk factors in the general adolescent population than adolescent-only interventions (20, 21). However, there is only one study targeting DEB that involves both adolescents with T1D and their parents/caregivers (22). Whilst this study demonstrated positive acceptability, feasibility and early efficacy (22), the clinical implementation of this study was not evaluated.

To date, in Australia there has been one study targeting prevention of ED risk factors in preadolescents (10–12 years) with T1D and their parents (23). This group-based intervention in preadolescents found improvements in self-esteem, body-esteem and reduction in thin-ideal internalization. No improvements were observed in glycaemic outcomes, and the exclusion of older adolescents leaves a significant gap in the literature, given their heightened risk for DEB. Therefore, despite promising results from international research trials including the DBP, there remains a gap in implementing evidence-based interventions tailored specifically to adolescents with T1D in Australia, and their families within routine clinical settings.

Building on previous DBP research, the primary aim of this study is to evaluate the feasibility and acceptability of implementing the DBP within a real-world Australian clinical setting, adapting the intervention for an adolescent-only population (age 13.0-17.9 years), and incorporating a novel parent/caregiver component. The secondary aim is to explore preliminary efficacy of the intervention in improving DEB, glycaemic outcomes, body appreciation and diabetes distress.

## Methods

### Study design and setting

This is a single-arm pilot study conducted at the John Hunter Children’s Hospital (JHCH) Diabetes service between 2025 and 2027. The JHCH diabetes service is a tertiary pediatric service in metropolitan NSW, Australia. The JHCH multidisciplinary diabetes team provides care to approximately 380 children and adolescents with T1D. Approval for this study was given by the Hunter New England Research Ethics Committee (2025/ETH01757) and registered with the Australian New Zealand Clinical Trials Registry (ACTRN12625001074482p). The protocol is reported in accordance with SPIRIT guidelines (24).

### Participants and recruitment

The study aims to recruit female adolescents aged 13.0–17.9 years with a diagnosis of T1D greater than 6 months, attending the JHCH Diabetes clinic. A pragmatic sample size of n=24 participants (representing approximately one third of the eligible population) will be recruited over a 12–18-month period. As the DBP intervention is designed to be delivered in groups of approximately 6 adolescents (17), this sample will enable delivery of four cohorts while enabling evaluation of feasibility and acceptability outcomes and the generation of preliminary estimates to inform future studies. Up to 24 parents or primary caregivers of recruited adolescents will also be recruited to participate in the additional parent component of the intervention. All participants will continue to receive usual care from the JHCH diabetes team alongside their participation in the study. Individuals with a clinically diagnosed ED, uncontrolled coeliac disease, or who are not proficient in speaking English will be excluded from the study.

The JHCH pediatric endocrine database (PED) will be used to screen adolescents for eligibility. Eligible adolescents, along with their parent/caregiver will be approached for participation face-to-face during routine clinic visits and provided with written study information and consent forms for consideration. Additional recruitment will occur via a digital flyer posted on the JHCH diabetes clinic’s Facebook page, a closed group to families attending the JHCH diabetes clinic. Parents who enquire via this method will be contacted by phone to confirm their adolescent’s eligibility, discuss the study, and provided with written information and consent forms for the adolescent and their parent.

Dual written consent will be obtained from both the adolescent and one parent or caregiver prior to enrolment and participants will be allocated to the next available DBP cohort. In line with the JHCH model of care, parent participation will be strongly encouraged; however, adolescents may still enrol independently to maximise participation. Recruitment commenced in October 2025.

### Facilitator training

Health care professionals from JHCH participated in 6 hours of DBP facilitator training provided by the DBP team at Oslo University Hospital, Norway. During the virtual training sessions, minor adaptations were made to language to ensure suitability for an exclusively adolescent Australian population. Following the training, session content was further reviewed and adapted by the JHCH dietitian, social worker and diabetes nurse educator to ensure use of age-appropriate language to maximise participant engagement and understanding. Activities that asked participants to utilise social media were excluded due to the Australian online Safety Amendment, legally restricting under 16-year-olds from holding certain social media platforms (25). An alternative activity was included that asked teens to find an inspirational public figure with T1D or someone who embraces body diversity and authenticity.

Two parent/caregiver sessions were developed by the research team to align with the JHCH Success With Individualised Insulin Management (SWIIM) program, which emphasises family involvement, communication and empowerment in diabetes management (26). Two one-hour sessions were designed to support and extend the adolescents learning and to address the role of parents in shaping body image and eating behaviors (21).

The parent/caregiver session content (shown in **Table 1**) was informed by a review of the literature on parental involvement in disordered eating interventions for adolescents with (22, 27) and without T1D (20, 21, 28); as well as research on parent influences on body image in adolescents with and without T1D body image (29–31). In addition to this literature, parent/caregiver questions and feedback during recruitment and discussion about the proposed program informed session content.

**Table 1:** Diabetes body project and parent/caregiver session overview.

Week	Participant group	Session topics
1	Adolescent	Define the thin ideal and where it comes from.
	Parent/Caregiver	Overview of the Diabetes Body Project, influences of body image in adolescents, body image and T1D, creating a supportive home environment, role plays, question on what each parent hopes they and their adolescent take away from the program.
	Between-session activity	1. Letter to a younger girl with T1D 2. Mirror body acceptance exercise
2	Adolescent	Examine the negative impacts of chasing the thin ideal
	Between-session activity	1. Rewind response letter 2. Top 10 list of things to do to resist thin ideal
3	Adolescent	Explore ways to resist pressures to be thin
	Between-session activity	1. Body image behavioral challenge 2. Body activism – choose an activity from week 2 top 10 list to do during the week 3. Write advice to younger self to reduce body image concerns
4	Adolescent	Discuss how to challenge our body image concerns
	Between-session activity	1. Social comparison 2. Social media and alternatives to social media
5	Adolescent	Learn and develop strategies to promote positive body talk
	Between-session activity	1. Advice for a good life with T1D 2. Tribute to insulin – creative piece on the positive aspects of insulin
6	Adolescent	Discuss and explore strategies to promote a positive relationship with diabetes and insulin
	Parent/Caregiver	Program feedback, factors that influence positive body image, healthy body image, communication strategies to support adolescents body image and T1D including practical role plays, group discussion on how parents feel they may be able to support teen differently.
	Between-session activity	1. Self- affirmation 2. Body activism

### Intervention

The adolescent intervention follows the manualized DBP developed by the original DBP team and has previously been described (17), with the addition of the parent/caregiver sessions developed for the current study. The DBP is adapted from the Body project and is grounded in cognitive dissonance theory, where participants actively critique the thin ideal through verbal, written and behavioral exercises (17, 19). Four DBP cohorts, each consisting of 5–6 adolescents, will participate together in 6 weekly 1-hour virtual group sessions facilitated by a diabetes nurse educator and diabetes dietitian from JHCH. Sessions follow the structured DBP facilitator manual and incorporate group discussion where participants explore the negative effects of pursuing the thin ideal, learn ways to challenge unhelpful body image thoughts, and discuss the benefits of insulin and diabetes self-care (see **Table 1**). Between-session activities are designed to re-enforce the cognitive dissonance-based session content (see **Table 1**). Further detail regarding the development and theoretical basis of the DBP is provided in previous publications (17, 32). Group sessions will be held outside of school hours to minimize disruption to school and family commitments. A catch-up phone call session will be offered for any missed sessions as per DBP protocol (17). The parent/caregiver sessions will be delivered by the same diabetes dietitian and the diabetes social worker from JHCH and will be facilitated for 1 hour in week 1 and week 6 of each program.

## Data collection and outcome measures

### Baseline

Baseline demographic and clinical data including age, Aboriginal and Torres Strait Islander status, duration of diabetes, HbA1c, 14-day average glucose %TIR, Insulin regime, Total daily dose (units/kg) and BMI Z score will be collected from medical records 2 weeks prior to group commencement.

### Primary outcomes

#### Feasibility and acceptability outcomes

1. Feasibility will be assessed by recording the proportion of eligible adolescents who enrolled in the program and parent/caregiver uptake. Attendance will be recorded for each of the 6 sessions. A session will be considered attended if the participant attends the virtual group session or engages in the catch-up phone call following the missed virtual group session. Attendance of at least 5 of the 6 session (80%) will be used as a feasibility threshold. Additionally, the format of attendance (virtual group or catch-up phone call) will also be recorded to provide additional information regarding feasibility of intended virtual group delivery. Between-session activity completion will be recorded for each participant. Completion of at least 10 of the 13 between-session activities (75%) will be used as a feasibility threshold.
2. Acceptability will be assessed through participant evaluation surveys. A separate adolescent and parent evaluation survey was developed specifically for the study to assess participants’ experience, perceived impact, and satisfaction with the program’s delivery and structure. Each survey includes a combination of 5-point Likert-scale items (“strongly agree” to “strongly disagree”) to capture quantitative data, along with open-ended questions to gather qualitative insights for program improvement and to identify future support needs.

### Secondary outcomes

1. Change in disordered eating behaviors, assessed using The Diabetes Eating Problem Survey – Revised (DEPS-R). The DEPS-R is a validated 16-item diabetes specific self-report measure of disordered eating. Higher DEPS-R scores are associated with increased DEB with a score of >20 indicating the need for further clinical investigation (33).
2. Change in glycaemic outcomes. HbA1c will be taken during routine clinic appointments and 14-day average glucose %TIR, downloaded from online clinic platform.
3. Change in body image assessed using the Body appreciation scale-2, (BAS-2). BAS-2 is a 10-item survey measuring body appreciation and positive body image. Responses are scored on a 5-point Likert scale, and higher scores indicate greater body appreciation (34).
4. Change in diabetes distress, assessed using the Problem Areas in Diabetes Survey – Teen version, (PAID-T) a 14-item survey to assess diabetes-related distress. Responses are scored on a 6-point Likert scale, and higher scores indicate higher perceived burden with the following cut points of diabetes distress: none-to-mild (<70), moderate (70–90), and high (>90) (35).
5. Change in body mass (BMI Z score). Height and weight measurements will be taken during routine clinic appointments.

Surveys will be generated using REDCap (36) and sent to participants via a secure email link. (See **Table 2** for data collection timepoints).

**Table 2:** DBP data collection timeline. .

Measure	Pre Program	Program Intervention						Post program	3 months post program	6 months post program
		Week 1	Week 2	Week 3	Week 4	Week 5	Week 6			
Age	✓									
Aboriginal and Torres Strait Islander status	✓									
Diabetes Duration	✓									
Insulin Regime	✓							✓	✓	✓
DEPS-R	✓							✓	✓	✓
BAS-2	✓							✓	✓	✓
PAID-T	✓							✓	✓	✓
HbA1c	✓								✓	✓
14-day average glucose %TIR	✓							✓	✓	✓
BMI Z Score	✓								✓	✓
Attendance (adolescent)		✓	✓	✓	✓	✓	✓			
Attendance (parent/caregiver)		✓					✓			
Between-session activity completion			✓	✓	✓	✓	✓	✓		
Adolescent evaluation								✓		
Parent/caregiver evaluation								✓		

### Data collection and storage

All data will be stored in a REDCap database administered by the Hunter Medical Research Institute. Any potentially identifiable information obtained for this research project will be treated as confidential and securely stored. Information will be coded and de-identified as necessary. Data will be available only to primary researchers and statisticians.

### Statistical analyses

Participant characteristics will be summarised as mean and standard deviation (SD) for the continuous variables (or median and IQR as appropriate), and frequency count and percentage for the categorical variables. Statistical analyses are planned in accordance with recommendations for pilot and feasibility studies, with emphasis on estimation and 95% confidence intervals (CIs) (37, 38).

### Feasibility

Proportions and 95% CIs will be reported for the proportion of eligible participants who enrol, participants who complete the program, between-session activity completion and parents/caregiver uptake and attendance. The proportion of the participants meeting attendance threshold of ≥80% will be reported, along with the format of attendance (virtual group or catch-up phone call) to provide additional information regarding feasibility of intended virtual group delivery. The proportion of participants meeting the between-session activities completion threshold of ≥75% will be reported, along with the completion rates of each of the 13 between-session activities. Values below these thresholds will not directly indicate if the intervention is feasible but rather indicate a possible need for redesign of certain program aspects of the intervention to better engage the adolescent population.

### Acceptability

Participant and parent/caregiver satisfaction survey data will be analyzed using descriptive statistics to summarise responses related to program acceptability, feasibility, and perceived impact. Frequencies and percentages will be reported for categorical variables (e.g., satisfaction ratings). Qualitative responses from open-ended questions will be analyzed using content analysis to identify common themes, insights, and suggestions for improvement (39).

### Preliminary efficacy

Within-person changes in DEPS-R score from baseline to each follow-up time point will be estimated and reported alongside 95% CIs. Within-person changes in HbA1c, glucose %TIR, BMI Z score, BAS-2 scores and PAID-T scores from baseline to each follow-up time point will be estimated and reported alongside 95% CIs.

As an initial investigation of the preliminary efficacy of the program, associations between change in DEPS-R score with each HbA1c, glucose %TIR, BMI Z score, BAS-2 and PAID-T will be explored using scatter plots and linear regressions. Regression results will be presented as slope estimates with 95% CIs.

## Discussion

Despite high rates of DEB and ED in adolescence with T1D and the associated risks of poorer glycaemic outcomes, diabetic ketoacidosis, and increased mortality (2–4, 6–8) ED prevention interventions in adolescents remain limited, especially within routine clinical care (13, 14).

This pilot study aims to address this critical gap by evaluating the implementation of an evidence-informed intervention (DBP) within a real-world clinical service.

This study includes several innovative elements. Recruitment will be clinic based, using routine clinic appointments to engage adolescents. By targeting younger adolescents (from 13 years) this study also involves prevention during a critical developmental window, prior to the peak onset of disordered eating (14–19 years) (10). This study will help to determine whether concepts of the DBP (e.g. critique of the thin ideal) are developmentally appropriate and translate to younger ages or require adaptations.

Finally, this study examines the feasibility of implementation within routine care, including recruitment, attendance, between-session activity completion, parent/caregiver uptake and acceptability. As a pilot study, estimates of change in clinical outcomes will primarily be used to inform the design and sample size considerations of future controlled trials. These outcomes will be considered collectively to provide information regarding participant engagement and potential barriers and facilitators to delivery, which is needed to inform the design and implementation of future larger scales in clinical settings.

### Strengths and limitations

Strengths of this study include the evaluation of the delivery by existing multidisciplinary diabetes clinicians, who have participated in brief facilitator training, demonstrating the feasibility of clinician-led delivery and integration into clinical practice. The study also extends previous DBP research by focusing exclusively on adolescents with T1D, a population at high risk of disordered eating behaviors. Furthermore, the addition of a parent/caregiver component is a key strength addressing a gap in research.

Limitations include the small, single-site design and lack of control group. As a result, findings relating to clinical and psychosocial outcomes should be interpreted as preliminary and intended to inform the design of future controlled trials. Nonetheless, this study provides an important step toward embedding ED prevention within pediatric diabetes care. If positive changes are observed, future research should include multi-site trials across a range of locations (metropolitan, regional and rural) to evaluate broader implementation and effectiveness.

## Data Availability

The raw data supporting the conclusions of this article will be made available by the authors, without undue reservation.
